# High doses of ketamine to improve neuronal edema in subarachnoid hemorrhage: we should consider other undesirable organ targets

**DOI:** 10.1186/s13054-020-03004-3

**Published:** 2020-06-18

**Authors:** Patrick M. Honore, Aude Mugisha, Luc Kugener, Sebastien Redant, Rachid Attou, Andrea Gallerani, David De Bels

**Affiliations:** ICU Department, Centre Hospitalier Universitaire Brugmann-Brugmann University Hospital, Place Van Gehuchtenplein, 4, 1020 Brussels, Belgium

We read with interest the recent article by Santos et al. regarding the use of S-ketamine to decrease spreading depolarizations and improve cytotoxic neuronal edema in patients with aneurysmal subarachnoid hemorrhage [1]. They note that the active enantiomer S(+) ketamine is two times stronger than the racemic form and four times stronger than the R(−) enantiomer [1]. Further, they report that the upper therapeutically recommended dose of S-ketamine for sedation is 2 mg/kg BW/h, but if deemed necessary, neuro-intensivists are using doses of up to 7 mg/kg BW/h. We would like to make some comments. S-ketamine is a non-competitive *N*-methyl-d-aspartate (NMDA) receptor ion channel blocker [2]. Its action is not limited to the brain, and as intensivists, we need to be mindful of the effects of ketamine upon other organs, especially when ketamine is used in high doses. Negative effects of these high doses have been described in the kidney [2, 3]. Renal medullary oxygenation is strictly balanced by a series of control mechanisms, which match regional oxygen supply and consumption [2]. Failure of these controls renders the outer medullary region susceptible to acute or repeated episodes of hypoxic injury, which may lead to acute tubular necrosis (ATN), especially of the thick ascending limbs [2]. In the kidney, any increase in circulating catecholamines (especial1y epinephrine) will cause vasoconstriction via the alpha-receptors and activation of the renin-angiotensin system [2]. As a result, despite normal renal blood flow, intramedullary ischemia may occur [2]. Increases in sympathomimetic hormones lead to renal cortical vasoconstriction, which is a compensatory attempt by the body to redistribute blood flow to the renal medulla, but in fact, it causes ischemia [2, 4]. S(+)-ketamine is about three to four times more potent as an anesthetic than R(−)-ketamine, but an equipotent dose of S(+)-ketamine alone may show weaker norepinephrine and serotonin uptake inhibition than R(−)-ketamine at anesthetic effective doses [2]. In a rat model of ischemia reperfusion of the kidney, high doses of S(+)-ketamine induced a higher score of histological changes, with the rise in catecholamine blood concentration thought to be the likely cause [2]. Further studies are needed to investigate the relationship between high doses of ketamine and the risk of acute kidney injury (AKI). In the study of Santos et al., it would be interesting to know the data regarding the rate of AKI between the control group and the high-dose group.

## Authors’ response

### High doses of ketamine to improve neuronal edema in subarachnoid hemorrhage: preliminary clinical data do not suggest an adverse effect on renal function

Edgar Santos, Sebastian Major, Renán Sánchez-Porras, and Jens P. Dreier

Thank you for your kind interest in our work. Aneurysmal subarachnoid hemorrhage (aSAH) is indeed characterized by an impressive list of potential complications. Thus, aSAH is associated with ~ 20 relevant intracranial and > 30 extracranial complications [5]. On top of this, there are numerous side effects of a wide range of medications. For our retrospective cohort study, we identified 66 patients from a prospectively collected database using prespecified criteria (32 from the University of Heidelberg, recruited 2004–2014, and 34 from Charité–Universitätsmedizin Berlin, recruited 2005–2010) [1]. Laboratory values are readily available for the Berlin patients of whom 14 were treated with S-ketamine. The Acute Kidney Injury Network (AKIN) has defined AKI as abrupt (within 48 h) reduction in kidney function leading to an absolute increase in serum creatinine of ≥ 0.3 mg/dl, an increase in creatinine of ≥ 50%, or a reduction in urine output (< 0.5 ml/kg/h for > 6 h) [6]. For the requested exploratory analysis, we here consider creatinine during the first 45 days after the initial hemorrhage (in total, 417 values, 12 ± 9 tests per patient). Based on this, 4 of 34 patients (12%) developed AKI: 2 of 20 (10%) without S-ketamine, 1 of 5 (20%) with S-ketamine below, and 1 of 9 (11%) with S-ketamine > 2 mg/kg body weight (BW)/h. In the latter patient, high-dose S-ketamine and mannitol treatment started on day 8 when she developed a dilated left pupil, decorticate posturing, intracranial pressure (ICP) above 20 mmHg, and computed tomography-proven transtentorial herniation. Serum creatine increased from 0.4 to 0.9 mg/dl between days 8 and 10 with full recovery thereafter. In parallel, a urinary tract infection with *Escherichia coli* was found and treated with ceftriaxone. In addition, the patient also suffered from arterial hypertension and systemic lupus erythematodes so that at least 5 potential etiologies of kidney damage coincided. In the follow-up (day 194), she was fully oriented but showed hemiparesis and mild aphasia (extended Glasgow Outcome Scale: upper severe disability). In the 9 patients, S-ketamine > 2 mg/kg BW/h did not lead to significant creatinine changes overall (before S-ketamine, 0.65 ± 0.17 mg/dl; during high-dose S-ketamine, 0.68 ± 0.18 mg/dl; paired *t* test; *P* = 0.443). It should be taken into account that ketamine, in addition to its potentially beneficial effects on spreading depolarizations [7], also leads to reduction in ICP and to savings in catecholamines with potential kidney protection [8]. Nonetheless, although our data do not suggest that S-ketamine increases the risk for AKI, renal function should be closely monitored.

## Data Availability

Not applicable.

## Abbreviations

NDMA: *N*-Methyl-d-aspartate
ATN: Acute tubular necrosis
AKI: Acute kidney injury
